# To evaluate the impact of a nurse-led JIA education program on parental satisfaction regarding knowledge of JIA and its management

**DOI:** 10.1186/1546-0096-9-S1-O24

**Published:** 2011-09-14

**Authors:** J Jones, J Munro, T Williams, A Augustine, K Taylor, S Piper, R Allen, J Akikusa

**Affiliations:** 1 Royal Children’s Hospital Melbourne, Australia; 2 Monash Children’s Hospital Clayton Australia; 3 The Murdoch Childrens Research Institute Melbourne Australia

## Background

In 2005 the Arthritis and Musculoskeletal Quality Improvement Project (AMQuiP) at the Royal Children’s Hospital (RCH) and Monash Children’s Hospital found over 50% of families wanted more education about JIA. A nurse-led education program was established.

## Aim

To evaluate parental satisfaction with the JIA education provided as part of the new model of care for JIA at the RCH.

## Methods

The RCH Rheumatology Database was used to identify all children diagnosed with JIA at RCH in 2009 according to ILAR (2001).

The families of those who had undertaken education were invited to complete the questionnaire used previously in the AMQuiP project. The questionnaire surveyed satisfaction regarding information provided about:

∎ JIA

∎ Medications

∎ Self management

∎ Managing disease flares

∎ Support services

∎ Emotional management

∎ School management.

Improvement in satisfaction was determined by examining the change in the percent of respondents indicating a need for more education when compared with those in 2005.

## Results

Sixty children were diagnosed with JIA at RCH in 2009, 77% of these families who had undertaken JIA education were invited to complete the questionnaire used previously in the AMQuiP project. Completed questionnaires were received from 63% of families. There was a substantial improvement of between 22-48% in parental satisfaction in all education related domains. The greatest improvements were in the disease (38%) and treatment (42%) domains. The areas with the least improvement were related to self management (22%) and support services (26%).

## Conclusion

The inclusion of a nurse-led education program has substantially improved parental satisfaction regarding their knowledge of issues integral to caring for children with JIA.

